# Implementation of a publication strategy in the context of reporting biases. A case study based on new documents from Neurontin® litigation

**DOI:** 10.1186/1745-6215-13-136

**Published:** 2012-08-13

**Authors:** S Swaroop Vedula, Palko S Goldman, Ilyas J Rona, Thomas M Greene, Kay Dickersin

**Affiliations:** 1 Department of Epidemiology, Center for Clinical Trials, Johns Hopkins Bloomberg School of Public Health, 615 N. Wolfe Street, Mailroom W5010, Baltimore, MD, 21205, USA; 2 Greene LLP, One Liberty Square, Suite 1200, Boston, MA, 02109, USA

## Abstract

**Background:**

Previous studies have documented strategies to promote off-label use of drugs using journal publications and other means. Few studies have presented internal company communications that discussed financial reasons for manipulating the scholarly record related to off-label indications. The objective of this study was to build on previous studies to illustrate implementation of a publication strategy by the drug manufacturer for four off-label uses of gabapentin (Neurontin®, Pfizer, Inc.): migraine prophylaxis, treatment of bipolar disorders, neuropathic pain, and nociceptive pain.

**Methods:**

We included in this study internal company documents, email correspondence, memoranda, study protocols and reports that were made publicly available in 2008 as part of litigation brought by consumers and health insurers against Pfizer for fraudulent sales practices in its marketing of gabapentin (see http://pacer.mad.uscourts.gov/dc/cgi-bin/recentops.pl?filename=saris/pdf/ucl%20opinion.pdf for the Court’s findings).

We reviewed documents pertaining to 20 clinical trials, 12 of which were published. We categorized our observations related to reporting biases and linked them with topics covered in internal documents, that is, deciding what should and should not be published and how to spin the study findings (re-framing study results to explain away unfavorable findings or to emphasize favorable findings); and where and when findings should be published and by whom.

**Results:**

We present extracts from internal company marketing assessments recommending that Pfizer and Parke-Davis (Pfizer acquired Parke-Davis in 2000) adopt a publication strategy to conduct trials and disseminate trial findings for unapproved uses rather than an indication strategy to obtain regulatory approval. We show internal company email correspondence and documents revealing how publication content was influenced and spin was applied; how the company selected where trial findings would be presented or published; how publication of study results was delayed; and the role of ghost authorship.

**Conclusions:**

Taken together, the extracts we present from internal company documents illustrate implementation of a strategy at odds with unbiased study conduct and dissemination. Our findings suggest that Pfizer and Parke-Davis’s publication strategy had the potential to distort the scientific literature, and thus misinform healthcare decision-makers.

## Background

National agencies, including the Food and Drug Administration (FDA) in the United States (US) and the European Medicines Agency (EMA) in Europe, regulate the marketing of drugs and medical devices. In the US, a drug may only be marketed legally after it receives FDA approval. Once the drug has at least one FDA-approved use, physicians may prescribe it for other unapproved uses, based on their clinical judgment; this is referred to as off-label use. In general, marketing of drugs for off-label uses is illegal both in the US and Europe, although regulation has changed over the years [1,2].

Pharmaceutical companies have used various strategies within their legal rights to market drugs for off-label uses [3]. For example, current FDA guidance states that pharmaceutical companies may use peer-reviewed articles to disseminate evidence of a drug’s effectiveness for off-label uses, as long as certain conditions are met [4]. Perhaps the most important of the conditions is that the information disseminated must not be false or misleading [4]. However, there is no regulation that all research findings must be published, and, therefore, a company may choose to selectively disseminate favorable findings. There is now ample evidence that selective reporting of study results, based on the strength and direction of findings (reporting biases), is widely practiced by the pharmaceutical industry [5,6].

Gabapentin (Neurontin®, Pfizer, Inc.) was first approved by the FDA for marketing in the US in 1993 as an adjunctive therapy for epilepsy in adults. Subsequently, the drug was approved for use as an adjunctive therapy for epilepsy in children (2000) and for the management of postherpetic neuralgia (2002). Gabapentin was initially marketed by Warner-Lambert’s Parke-Davis division, which was later acquired by Pfizer (thus, hereafter referred to as Pfizer and Parke-Davis).

Shortly after gabapentin's initial FDA approval for use in epilepsy, Pfizer and Parke-Davis conducted a series of “marketing assessments” for several off-label uses of the drug. The “marketing assessments” for each off-label indication provided a detailed analysis of the importance of the clinical condition, current treatment alternatives, market share commanded by each intervention, and a forecast of financial profits associated with two alternatives that were at the company’s disposal to market the drug for each indication — either obtaining FDA approval for marketing associated with a given indication (an indication strategy) or adopting a publication strategy (these are the terms used in the “marketing assessments”). The goal of the publication strategy was to conduct clinical trials and ‘to disseminate the information as widely as possible through the world’s medical literature’ [7].

To date, internal company documents related to gabapentin have been made publicly available through two separate legal actions. Documents from a whistleblower-initiated government lawsuit involving violation of off-label marketing regulations, settled in 2004 (hereafter referred to as the 2004 whistleblower litigation), have previously been described [8]. In addition, a large number of previously undisclosed internal company documents were made available through subsequent litigation, brought by consumers and health insurers that paid for prescriptions of gabapentin, involving allegations of fraud (In re Neurontin Marketing and Sales Practices Litigation, MDL 1629, Civil Action NO. 04-cv-10981-PBS, United States District Court, District of Massachusetts) [9]. Documents related to this litigation (hereafter referred to as the 2008 consumers and health insurers litigation) first became publicly available in 2008. While internal company documents made available in the 2004 whistleblower litigation and the 2008 consumers and health insurers litigation contain overlapping and unique information, the 2008 documents contain considerably new information. Both sources were used for research presented here and in a previous study [10]. The documents covered by these two lawsuits provided us with a unique longitudinal view of company deliberations and decisions concerning marketing of one of its drugs. We distilled the literally thousands of pages of new and older information into themes of interest to those who study clinical trial conduct and integrity.

The objective of the current study was to describe the implementation of a publication strategy for off-label marketing of gabapentin, within the context of reporting biases and spin of Pfizer and Parke-Davis’s clinical trial findings, for four off-label uses: migraine prophylaxis, treatment of bipolar disorders, neuropathic pain, and nociceptive pain. We use snapshots of internal company documents to illustrate our findings.

## Methods

Source documents for this study were provided to the senior author (KD) for preparation of an expert report for the plaintiffs’ lawyers in 2008 as part of the litigation brought by consumers and health insurers against Pfizer. We included information about all trials sponsored by Pfizer and Parke-Davis and documents relating to the four off-label uses revealed by the company during the discovery process that accompanied the litigation. Through communications with counsel involved in the litigation, Pfizer agreed to waive any confidentiality claims concerning all documents examined as part of KD’s expert report. In addition, we retrieved internal company documents that were made publicly available in the Drug Industry Documents Archive (DIDA; http://dida.library.ucsf.edu) following the 2004 whistleblower litigation [8]. In general, full versions of examples of internal company documents shown in the Additional file 1 may be accessed from DIDA using the identification number on the bottom of the relevant page. We redacted the names of individuals in the internal company documents provided in the Additional file 1 and excerpts shown in the manuscript. In one case, however, we did not redact the individual’s name since the company personnel referred to the study using the individual’s (principal investigator’s) name and not the study number.

We examined marketing assessments prepared by or for the company for each of the four off-label uses of gabapentin (migraine prophylaxis, treatment of bipolar disorders, neuropathic pain, and nociceptive pain) to identify discussion of a publication strategy as a possible marketing option. We examined over 20,000 pages of internal company communications, memos, and other documents to identify the definition of a publication strategy and details related to its implementation. We also examined the documents for evidence of reporting biases (dissemination of research findings influenced by the nature and direction of results) within our sample of clinical trials.

To identify reporting biases, we compared internal company protocols and research reports with the main publication, if one existed, for each study included in our analysis. The internal company research reports were prepared for or by Pfizer and Parke-Davis at the end of each study and provide documentation of the methods for the trial and results of analyses. Typically, the internal company research reports also included as appendices the study protocol and its amendments, the analysis plan, and output from statistical analyses. We selected one main publication for each study in the following order of priority: full-length publication, letter to the editor, non-systematic review showing a pooled analysis of selected trials, and a conference abstract [10]. We assessed the documents for appearance of the following reporting biases: (a) publication bias (that is, publishing a study report based on strength and direction of findings), (b) outcome reporting bias (that is, selective reporting of outcomes based on the strength and direction of findings), (c) location bias (that is, in this study, reporting in journals with lower or higher circulation, based on findings), and (d) time lag bias (that is, timing of publication based on the strength and direction of findings) [6,11]. Publication bias and outcome reporting bias in the context of the documents we examined for this study have been explicitly explored in a previous article and are not discussed here [10].

In this study, we also examined control of what was published, assessed the documents for evidence of ghost authorship (that is, failure to report as an author individuals who have made a substantial contribution to the study or the article) [12], and assessed spin of the study findings. We considered spin to exist when we observed either an explicit description of spinning study findings in the internal company documents or a description in the main publication that appeared to re-frame the study results in order to explain away unfavorable findings or to emphasize favorable findings. Our definition of spin was derived from terminology used in internal company documents and other research related to spin [13].

We obtained data on circulation of journals publishing the main publication for studies from internal company documents (dated November 28, 2001) for 10/12 trials (see Additional file 1: Figure S9 for an example). For the remaining two trials, we obtained the 2001 journal circulation from other sources: in one case from the 2001 edition of the *Ulrich’s Periodicals Directory*[14], and in the other through communication with the publisher. To check for validity of the data retrieved from *Ulrich’s*, we compared the circulations in the internal company documents with those in *Ulrich’s*. In general, the journal circulation data were comparable, though not identical.

We examined internal company research reports and published reports for each clinical trial and considered the direction of trial findings to be statistically significant if the primary outcome(s) was reported to be statistically significant or if the *P* value was less than 0.05. If more than one primary outcome was described in the reports or if there was no distinction between primary and secondary outcomes, we selected the smallest *P* value favoring gabapentin to determine statistical significance of findings in the trial. To determine the time to publication for each trial, we calculated the time in months between the date issued written on the internal company research report and the date of the published report. For estimating the time to publication, we did not include trials where an internal company research report was not available (n = 2) or where the date of issue was not available (n = 1).

In preparation for litigation and the jury trial, all authors reviewed all documents utilized for this paper. Assessments of reporting biases, spin, and journal circulation were made by SV and KD and discrepancies in the assessments between the two authors were resolved through discussion and reference to the original documents. All authors discussed the findings and agreed upon their interpretation.

We present narrative description of events, counts, and snapshots to depict reporting biases and we illustrate our findings from relevant internal company documents.

## Results

We identified a total of 21 trials sponsored by Pfizer and Parke-Davis (three trials each for migraine prophylaxis and bipolar disorders, nine trials for neuropathic pain, and six trials for nociceptive pain), of which 13 were associated with a published report [15-27]. We excluded one published trial of gabapentin for neuropathic pain since we had access to no internal company documents related to this trial [18]. One trial each for migraine prophylaxis and treatment of neuropathic pain and all 6 trials for nociceptive pain were never published [10].

### Publication strategy

**Figure 1:**
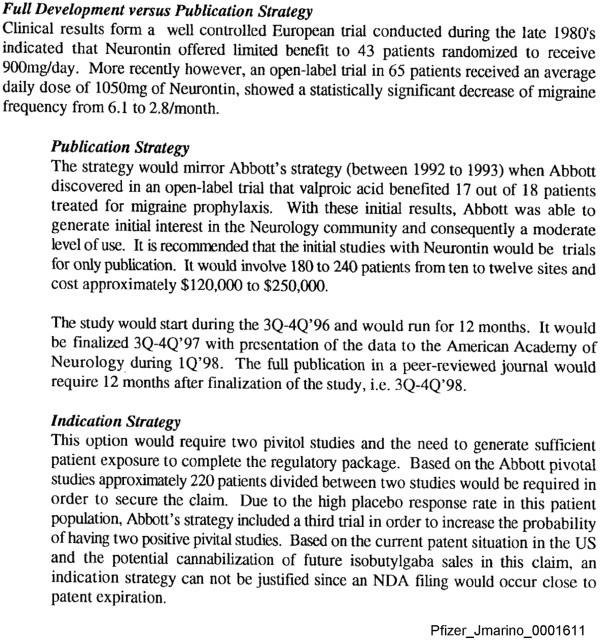
**Publication strategy versus indication strategy.** A publication strategy consisted of conducting clinical trials with an objective of disseminating results and generating clinical use of the drug for an unapproved indication, as opposed to obtaining FDA approval for marketing (that is, an indication strategy). The figure is a snapshot of a “marketing assessment” of gabapentin for migraine prophylaxis illustrating recommendation of a publication strategy that consisted of conducting trials to disseminate results and generating clinical use of the drug for an unapproved indication instead of obtaining FDA approval (that is, an indication strategy). Excerpted from Pfizer_JMarino_0001611 [28].

For each of the four off-label indications we included in our study, a document titled ‘marketing assessment,’ designed to examine the financial impact of seeking FDA approval for a new indication versus other methods of increasing sales for the indication, preceded clinical trials sponsored by Pfizer and Parke-Davis. Our examination of internal company ‘marketing assessments’ for bipolar disorders and neuropathic pain revealed clear recommendations to adopt a publication strategy as opposed to an indication strategy, that is, conducting trials with an aim to obtain FDA approval for the indication; this finding has been reported previously for these two indications [8]. The ‘marketing assessment’ for migraine prophylaxis, dated July 31, 1996, also recommended a publication strategy (see Additional file 1: Figure S1). Specifically, the recommendation was to ‘conduct only publication study(ies) in the U.S.,’ and in a manner comparable to what was described for bipolar disorders and neuropathic pain, to publish the results, ‘if positive’. The migraine prophylaxis ‘marketing assessment’ also stated ‘an indication strategy cannot be justified since an NDA (New Drug Application) filing would occur close to patent expiration’ (Figure 1).

The ‘marketing assessment’ for nociceptive pain, dated December 1998, recommends that no attempt to extend the patent for gabapentin should be made, in part because the current focus was on development of a new drug, pregabalin. The document describes that the primary objective of any new trials should be to demonstrate efficacy and that data on secondary endpoints, such as gastrointestinal tolerance, ‘would be for publication only’ (see Additional file 1: Figure S2).

### Implementation of the publication strategy and biased reporting

#### Controlling the message -- what should be reported?

Internal company documents including correspondence among company employees indicate that to achieve its marketing goals, Pfizer and Parke-Davis appear to have exerted control over the message delivered in published clinical trial results. One approach to controlling the message, that we previously reported, appears to have been not reporting, or selectively reporting, negative trial findings (publication bias and outcome reporting bias) [10].

**Figure 2:**
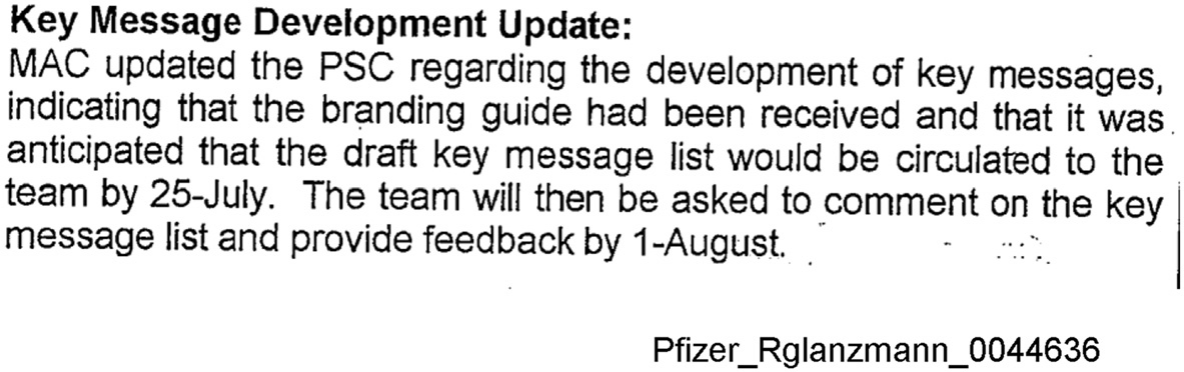
**Key messages for inclusion in publications appeared to have been developed based on a branding guide.** Excerpt from internal company minutes of meeting with Medical Action Communications (MAC) and the Neurontin Publication Sub-committee (NTN PSC) dated July 18, 2011. The document illustrates how “key messages” appeared to have been developed by MAC based on a branding guide for gabapentin. Excerpted from Pfizer_RGlanzmann_0044636 [29].

A Neurontin Publications Subcommittee (NTN PSC) was formed within Pfizer and Parke-Davis to implement a publication plan. Minutes from meetings between the NTN PSC and Medical Action Communications (MAC), a medical writing company, indicate that a list of key messages, guiding the content of published reports related to the trials of gabapentin for off-label indications, was developed based on a branding guide (referred to in Figure 2 and in Additional file 1: Figure S3).

**Figure 3:**
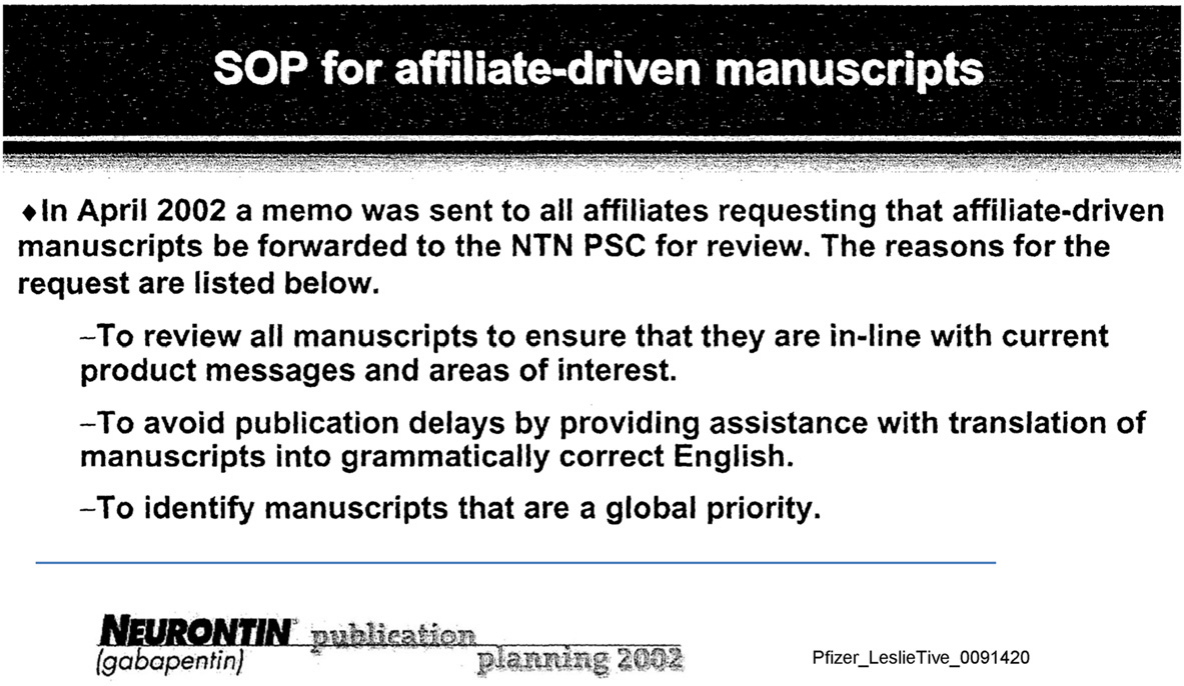
**All affiliate-driven manuscripts were to be reviewed by the Neurontin Publication Sub-committee to ensure the content was consistent with current product messages.** This figure shows an excerpt from an internal company document related to the Standard Operating Procedure (SOP) for manuscripts from company affiliates, indicating objectives for review by the Neurontin Publication Sub-committee (NTN PSC). Excerpted from Pfizer_LeslieTive_0091420 [30].

A standard operating procedure related to publication of affiliate-driven manuscripts was identified in internal company documents dated October 16, 2002, and it sheds further light on the publication planning process (see Figure 3). (The term affiliate in this context refers to Pfizer’s foreign affiliates, that is, corporations related to Pfizer by either shareholdings or other means of control, including subsidiary, parent, or sibling corporations). According to the internal company documents, “affiliate-driven manuscripts” were written for Pfizer and Parke-Davis by MAC and sent to the authors for approval. Each article was coordinated by a manuscript team, consisting of representatives from the medical and marketing divisions of the company. The documents also indicate that all affiliate-driven manuscripts should be forwarded to the NTN PSC for review. One of the objectives of manuscripts being reviewed by the NTN PSC was to ‘ensure that they are in-line with current product messages and areas of interest’ (see Figure 3 and Additional file 1: Figure S4).

##### Example of how Pfizer and Parke-Davis controlled what should be reported -- study 945–224

Negative findings from Study 945–224 were documented in an internal company research report dated February 7, 2000. A manuscript describing the study findings was first submitted to *Diabetic Medicine* on March 11, 2002 and after it was rejected, to *Diabetologia* on October 22, 2002. Peer reviewers for both journals expressed concerns related to the methodological approach, inconsistency of findings from Study 945–224 with findings from other studies of gabapentin for neuropathic pain, as well as company bias (see Additional file 1: Figure S5).

Before the negative reviews and rejection of the manuscript describing findings from Study 945–224, MAC drafted a review article dated November 11, 2001, titled ‘Gabapentin Dosing for Neuropathic Pain.’ The draft, accompanied by a document titled ‘Key messages included in this manuscript’, was sent to Pfizer (see Additional file 1: Figure S6). The key messages related to the article focused on: (1) highlighting the adverse event profile for other medications used in neuropathic pain in contrast to the ‘excellent safety and tolerability record of gabapentin’ and (2) recommendations for dosing, up to 3,600 mg/day. The published version of the review, dated January 2003, included data from Study 945–224 in a selective, pooled analysis of data from five trials sponsored by Pfizer and Parke-Davis, and claimed an overall benefit of gabapentin for neuropathic pain [16]. The key messages outlined for Pfizer and Parke-Davis by MAC were included in the review article. MAC’s contribution was not acknowledged in the published article [16], a practice consistent with the definition of ghost authorship.

#### Controlling the message -- what should be reported?

##### Spin

**Figure 4:**
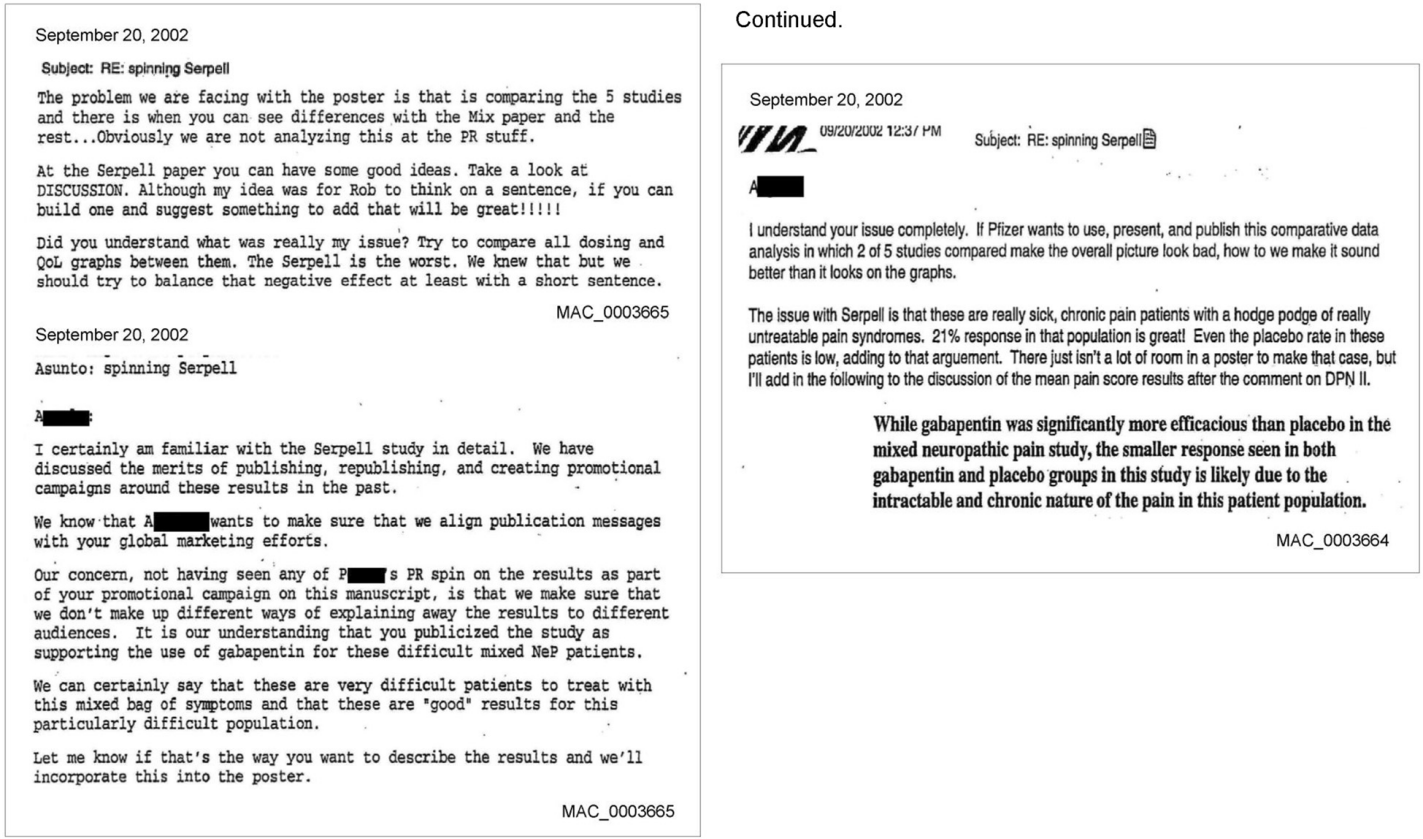
**Spinning Serpell - Excerpts from internal company emails illustrating decisions to spin presentation of findings from Study 945–306.** Excerpts from internal company emails illustrating the decision to spin presentation of findings from Study 945–306. Dr. Serpell was the first author of the trial publication corresponding to Study 945–306. Excerpted from MAC_0003664 [31] and MAC_0003665 [31]. Other than Dr. Serpell’s, names and contact information of individuals have been redacted by us such that only first initials can be seen.

We identified spin in publications related to 8/12 trials included in our analysis (see Figure 4 and Additional file 1: Figure S7 and Additional file 1: Table S1). We classified the following as spin: emphasis in the published report on outcomes that were not specified in the study protocol (Study 879–201) [27]; conclusions that did not match study findings described in the internal company research report (Study 945–220) [22]; extensive rationale to explain away statistically non-significant (unfavorable to the sponsor) findings (Study 945–209; 945–291; No study number - Gorson) [21,23,25]; conclusion of treatment effectiveness from an uncontrolled study (Study 945–250) [26]; emphasis on statistically significant secondary outcomes despite negative findings for the primary outcome (Study 945–271) [20]; and an explicit description of an attempt to spin study findings (as described in internal company emails) (Study 945–306) [24].

##### Example of spin

We identified internal company emails related to Study 945–306 that discussed how the study findings would be presented in a poster [32], using spin to indicate findings favoring gabapentin. Indeed, the subject line for the email conversation was ‘spinning Serpell’ (Dr. Serpell was the lead study investigator). An excerpt from the email communications follows (see Figure 4 and Additional file 1: Figure S7 and Additional file 1: Table S1):

"‘*If Pfizer wants to use, present, and publish this comparative data analysis in which 2 of 5 studies compared make the overall picture look bad, how to (sic) we make it sound better than it looks on the graphs.*’"

#### Controlling the message -- where should the results be reported?

At the request of the NTN PSC, an extensive list of journals and scientific congresses was developed ‘for the Neurontin publications plan’ (see Additional file 1: Figure S8). Documents from a subsequent meeting of the NTN PSC, dated November 28, 2001, illustrate the profiles developed for 115 journals and 80 congresses across 11 medical specialties (see Additional file 1: Figures S9 and Additional file 1: Figure S10 and Additional file 1: Table S2). The documents profiled the journals and congresses in extensive detail, providing data on, for example, the types of unsolicited manuscripts accepted by a journal, percentage of primary manuscript acceptance, time from acceptance to publication, total publication time, whether and where the journal was indexed, impact factor, total circulation, target audience, and the percent geographic distribution of the journal. In the case of congresses, the detailed profiles included whether abstracts were accepted, satellite symposia were permitted, the target audience, geographic audience, total attendance, and the total professional attendance.

**Figure 5:**
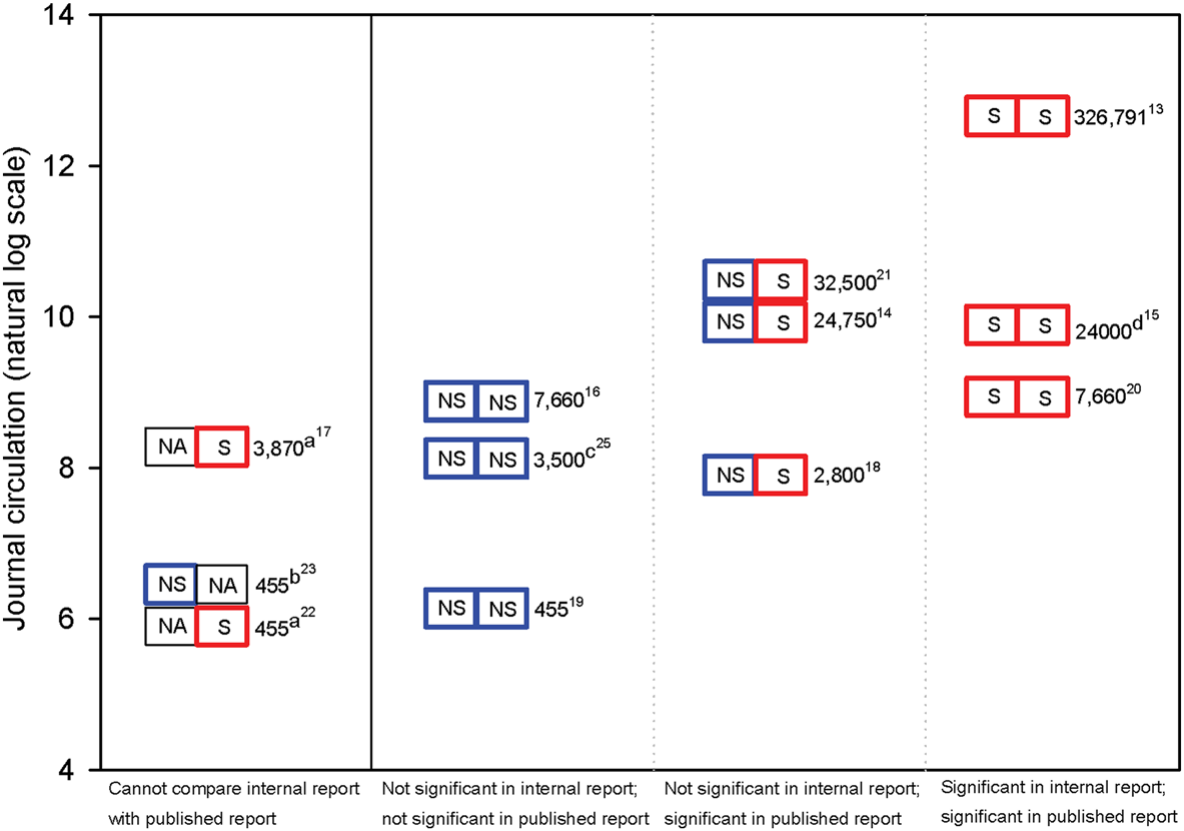
**Journal circulation of main publication, by statistical significance of primary outcomes in the internal company research report and the published report.** Each study is represented by a two-sided rectangular box which indicates the statistical significance of the primary outcome according to (1) internal company documents (left side, protocol-defined primary outcome[s]) and (2) the main publication (right side, publication-defined primary outcome[s]). The text to the right of the box represents the journal's circulation. Numerical superscript specifies the reference number for the main publication of the study. NA - Not available. S - Primary outcome(s) statistically significant. NS - Primary outcome(s) not statistically significant. a. Cannot compare statistical significance: Internal company research report was not available (Studies 945–250 and No study number - Gorson). b. Cannot compare statistical significance: Statistical significance not reported for any publication-specified primary outcome(s) (Study 879–201). c. Journal circulation obtained from *Ulrich's Periodicals Directory*. 2001 edition; Volume 3. Page 5018. Bowker, New York (Study 945–224). d. Journal circulation for 2001 obtained through communication with the publisher (Study 945–411).

Figure 5 illustrates the journal circulation, as listed in the internal company documents, compared to statistical significance of findings, for each published trial included in our study. Trials with statistically significant publication-specified primary outcomes were generally published in journals that had higher circulations than trials with publication-specified primary outcomes that were not statistically significant. Three studies (945–220, 945–291, and 945–276) with statistically non-significant findings for the protocol-specified primary outcome, subsequently reported publication-specified primary outcomes as statistically significant (inconsistent findings) [10], were published in journals with moderate circulations. That is, the size of journal circulation for reports with inconsistent findings was generally between the circulation of journals publishing consistently non-significant and consistently significant findings. Decisions about journal for publication may have been related to additional factors, such as the journal’s own selection criteria. For example, internal company documents related to peer review of Study 945–306, which was initially submitted to the *BMJ* (circulation listed as 115,000), revealed that the reviewers were concerned about ‘badging’ of the trial, in the context of the company’s employees serving as authors on the published report (see Additional file 1: Figure S11). Findings from this study were subsequently published in the journal *Pain* with a smaller circulation (listed as 7,660) [24].

#### Controlling the message - when should the results be reported?

Several internal company documents illustrated that personnel affiliated with the marketing division within Pfizer and Parke-Davis may have influenced the timing of publication of trial results. We present two examples for trials testing the effectiveness of gabapentin for neuropathic pain.

##### Example 1 of timing of publication

Study 945–224 was an international multicenter trial that compared three doses of gabapentin with placebo for the treatment of painful diabetic neuropathy. The trial was conducted between May 1998 and September 1999 and the internal company research report was issued on February 7, 2000, indicating statistically non-significant findings for the primary outcome. Internal company documents reveal that the decisions on whether and when to publish this trial’s results appear to have been made by marketing personnel (See affiliation of personnel in email communications shown in Additional file 1: Figure S12). It appears that the goal of marketing personnel was to publish the statistically significant results from two other trials the company sponsored (Study 945–306 and a study on postherpetic neuralgia that we did not include in our analysis [33]) before the statistically non-significant results from Study 945–224.

"‘*What is critical is that −224 is NOT submitted to any publication until we know WHEN the 2 UK studies are going to be published. This will allow us to ensure that 224 is not published before the UK studies.’ *[34]"

The decision regarding publishing findings from Study 945–224 appears to have been driven by marketing concerns, as illustrated by the following email communication by a Senior Marketing Manager:

"‘*By the way, C_______, from a MKT point of view we are not interested at all in having this paper published because it is negative!!! So don’t put this as a high priority in your list….’*[35]"

"*‘In fact it will be great to have it published by the end of 2004!!! Just for Pregabalinesiclaunch…’ *[35]"

**Figure 6:**
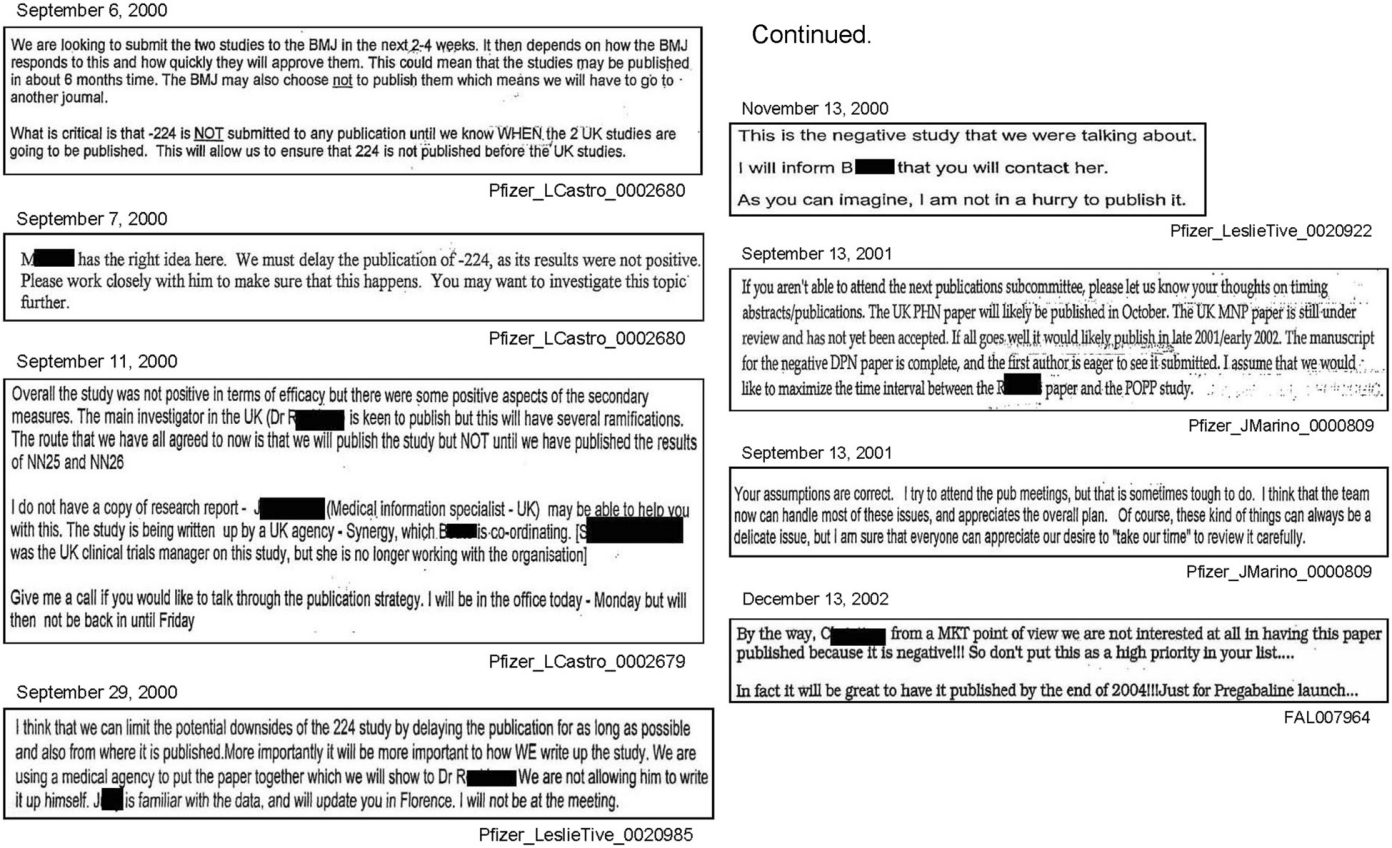
**Internal company emails illustrating decisions to delay publication of trial findings from Study 224 that were not statistically significant.** Excerpts from multiple internal company emails, chronologically arranged, illustrating the decision to delay publication of results from Study 945–224 with statistically non-significant findings. Individuals’ names have been redacted by us such that only first initials can be seen. Excerpted from Pfizer_LCastro_002679 [34], Pfizer_LCastro_002680 [34], Pfizer_LeslieTive_0020985 [37], Pfizer_LeslieTive_0020922 [38], Pfizer_JMarino_0000809 [39], and FAL007964 [35].

As noted earlier, summary findings from Study 945–224 were eventually described, in 2003, within a non-systematic review of other neuropathic pain trials [16]. The article described briefly each of the five trials it included, tabulated the mean change in pain scores for each comparison group within each trial, described in the text the statistical significance associated with the primary and secondary outcomes in each trial, and provided in the text a pooled analysis of all five trials. A detailed description of the evolution of the decision to publish results from Study 945–224 is available elsewhere [36]. See Figure 6 for excerpts from email conversations involving marketing personnel affiliated with Pfizer and Parke-Davis illustrating decision-making regarding publication of findings from Study 945–224.

##### Example 2 of timing of publication

Study 945–271 (referred to in the internal company documents as ‘POPP’) was a multicenter, crossover trial in Scandinavian countries that compared gabapentin with placebo for postoperative and post-traumatic neuralgia. The trial was conducted between November 1998 and November 2001 and the internal company research report was issued in March 2003, indicating statistically non-significant findings for the primary outcome.

**Figure 7:**
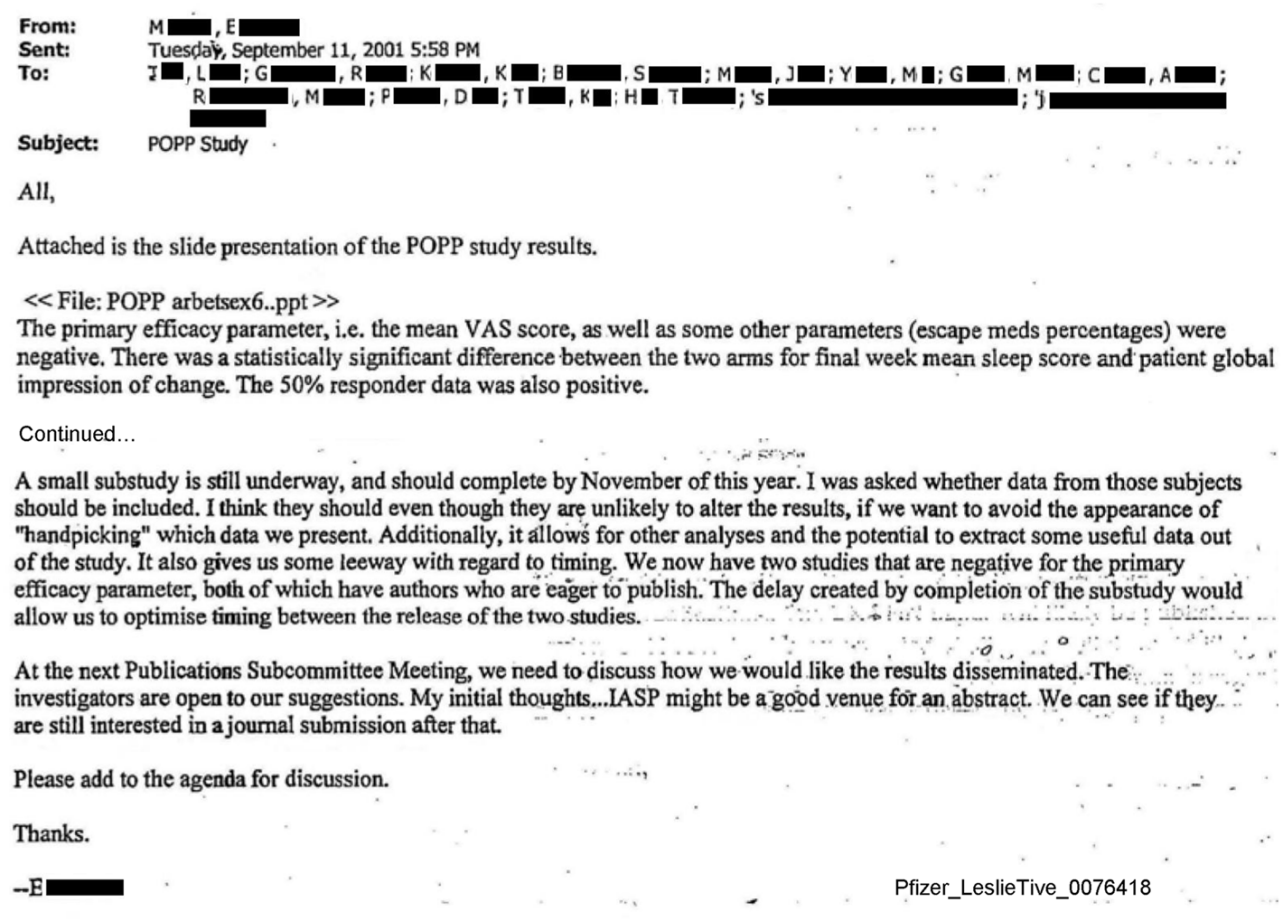
**Internal company emails illustrating decisions to delay publication of trial findings from Study 271 that were not statistically significant.** Excerpts from an internal company email illustrating decision to delay publication of results the ‘POPP’ study (Study 271 or Study 945–271) with statistically non-significant findings. Excerpted from Pfizer_LeslieTive_0076418 [40].

An internal company email conversation indicates that Pfizer and Parke-Davis personnel were concerned about publishing the statistically non-significant findings from Study 945–271 shortly before or after publishing statistically non-significant findings from another trial, Study 945–224 (see Figure 7).

"*‘The manuscript for the negative DPN paper is complete, and the first author is eager to see it submitted. I assume that we would like to maximize the time interval between the R_________* paper *and the POPP study.’*"

A different internal company email illustrates that although the primary investigator for Study 945–271 was keen on publishing the trial’s findings, the company sought to wait until a sub-study associated with Study 945–271 was completed in order to ‘optimise timing between the release of the two studies’ (see Figure 7 and Additional file 1: Figure S13). The findings from Study 945–271 were described in an internal company research report dated March 2003, although internal company documents indicate that Pfizer was aware of the statistically non-significant results from this trial at least by September 2001. The results from Study 945–271 were reported in full in August 2008, more than five years after the internal company research report, and nearly seven years after the results were available to the company [20,41].

A delay in publication greater than three years from the date of the internal company research report was observed for two other trials where the protocol-specified primary outcomes were not statistically significant per internal company documents (Studies 945–220 and 945–209) [22,23].

#### Controlling the message -- who should report?

##### Ghost authorship

Available internal company documents for two of the 12 included studies indicate that professional medical writers drafting the published reports were not appropriately acknowledged. Internal company documents indicate that personnel at MAC drafted and responded to the peer reviewer comments regarding the non-systematic review describing findings from five trials including Study 945–224 [42]. But MAC’s contribution was not acknowledged in the published report [16]. Similarly, the contribution of medical writers at Synergy Medical to the published manuscript for Study 945–306, described in internal company documents, was not acknowledged in the published report [24].

## Discussion

Parke Davis and Pfizer’s decision to use a publication strategy to market off-label uses of gabapentin was recognized by the courts in the 2004 whistleblower case, and this decision was documented in the biomedical literature in 2006 by an expert witness and his colleagues [8]. What is new in our study and in the 2008 consumers and insurers litigation is the discovery that various forms of reporting biases and spin were used in conjunction with the publication strategy. We previously reported failure to publish trials with statistically non-significant findings and outcome reporting bias [10]. In this study we also observed that publication occurred in journals with higher or lower circulations related to statistical significance of findings; delay in publishing statistically non-significant findings; tailoring of publication content to reflect key marketing messages; adding spin to scientific publications such that conclusions favoring gabapentin were emphasized and conclusions that did not favor gabapentin were explained away; and indicators of ghost authorship. Each form of bias, and spin, on its own, could be seen as a relatively minor issue. The value of our findings is in the overall picture that emerges from what appears to be the simultaneous use of many forms of reporting bias and spin, all within the context of a pharmaceutical company’s publication strategy, implemented for marketing purposes.

Our manuscript builds on previous research [8] by examining longitudinally the initial company decision to adopt a publication strategy for marketing gabapentin and increasing sales, and the consequences of that decision in terms of publication. Our work also provides a framework for distinguishing a publication plan from a publication strategy. A publication plan is typically employed by industry to manage publication of clinical trial findings [43,44]. In contrast, a publication strategy involves conducting clinical trials not for the purpose of obtaining regulatory approval to market for a condition but to publish the trial findings in order to market the drug for off-label uses.

Our definition for spin extends work previously done by others on this topic, which used only published literature and focused on trials with statistically non-significant findings [13]. Our findings provide a more detailed elucidation of strategies for spin, for example, emphasizing favorable findings from outcomes not pre-specified in the trial protocol or from statistically significant secondary outcomes, reaching conclusions that are not consistent with trial design or findings, using extensive rationale to explain away unfavorable findings, and in the case of one trial, explicitly describing attempts to spin trial findings (see Figure 4 and Additional file 1: Figure S7 and Additional file 1: Table S1).

Our study has limitations. Our findings in this study related to reporting biases are influenced by what we identified in the internal company documents provided through the legal discovery process. We include as authors of this article all persons involved in this aspect of the legal case, for transparency. We adopted a case study approach, whereby we described the findings from our observations instead of conducting a detailed, qualitative content analysis of the internal documents. We included in our analysis all available internal company documents. However, there may be other documents that are unavailable to us that could provide additional information regarding issues discussed in this article. We did not examine pharmaceutical trials on other topics or supported by other companies, nor did we examine trials sponsored by not-for-profit entities. Thus, we are unable to say that our findings are uniquely or generally related to industry funding. Indeed, reporting biases have been documented in trials sponsored by both industry and not-for-profit entities [45,46]. We did not conduct an exhaustive search for all conference abstracts related to each included trial. Our choice of the main publication for each trial may have affected our observations on reporting biases. However, our criteria for selecting the main publication were based on the published report that provided the most information among all available published reports of the trial. We cannot be certain that the reporting biases and spin we observed are a direct consequence of the discussion and actions reported in the internal company documents we examined. We did not examine study data for the number of prescriptions for off-label uses. Analyses presented as testimony in the 2008 consumers and health insurers litigation indicate that there was a general rise in the number of prescriptions for gabapentin over the time period covered by the publications examined in our study [47], although it is not possible to establish to what extent publications made an independent contribution to this increase.

On its own, a goal to disseminate research findings via the published literature is broadly applicable, whether the investigators are in academia, industry, government, or elsewhere. In the case of gabapentin, the internal company documents illustrate that as part of implementation of the publication strategy, the company’s marketing personnel appear to have controlled many aspects of publication and presentation of study results, including content (see Figure 7). Although trial investigators outside the company were frequently keen to publish study findings (including situations when tests of gabapentin’s effectiveness were not statistically significant), internal company emails indicate that decisions about the content and timing of journal publication may have been strongly influenced by the company’s marketing personnel.

Pharmaceutical companies have a strong financial incentive to pursue off-label marketing because such marketing can influence physicians’ prescribing practices and, consequently, the companies’ profits. Indeed, published reports of research studies play an important role in marketing by pharmaceutical companies. Documents released during litigation against Pfizer and Parke-Davis describe ideas discussed during training sessions for medical liaisons within Parke-Davis [48]:

"‘*Notice all the studies we talk about, nothing gets a doc more interested in a drug than a study*[48]*.*’"

Most clinical trials testing the effectiveness of drugs are sponsored by two types of entities: the pharmaceutical industry and not-for-profit organizations [49-51], such as the National Institutes of Health in the U.S. The primary motivation for conducting the trials is different for the two entities. The for-profit pharmaceutical industry has a legal fiduciary responsibility to its shareholders, with a corresponding goal of financial profit, achieved either through marketing approval by the FDA or through other marketing strategies. Not-for-profit organizations and their research partners in academia, on the other hand, generally conduct clinical trials in the context of scientific inquiry. The reporting biases we observed represent deliberate dissemination of promotional messages to market gabapentin [52]. This is not a situation likely unique to gabapentin and Pfizer and Parke-Davis [53-55]; rather, the opportunity to examine internal company documents has provided us with rare insight into industry communication and practices regarding trials included in our study.

The public relies on a regulatory approval system designed to protect them from the marketing of ineffective and potentially harmful drugs. The public is assured that for drugs approved by the FDA, an unbiased examination of safety and effectiveness for the approved indication has been conducted. There is no such system to ensure the safety and effectiveness of a drug used off-label. Because the FDA and public can typically access only the published reports of trials sponsored by a company for off-label uses of a drug, they have to rely on the published literature as a truthful and complete repository of the trial findings. When trials are conducted solely within a publication strategy framework, and the strategy includes reporting findings in a biased manner to further a company’s marketing goals, there is the potential for the scientific record to be distorted.

In addition to standard criminal and civil penalties, several other government policies are in place to hold companies accountable for unethical, off-label marketing practices and prevent future occurrences. Companies that repeatedly conduct clinical trials as part of a publication strategy and intentionally engage in unethical and biased reporting of trial findings could be suspended or even debarred from doing business with the federal government so as ‘to protect the public interest’ and ensure ‘the integrity of Federal programs’ [56]. Suspension or debarment should be a strong deterrent, because pharmaceutical companies rely for large portions of their revenue from these programs (for example, Medicare, Medicaid) and the sums involved are often far larger than the revenue earned by improperly marketing a single drug. The government may also suspend or debar individual corporate employees [57], making them unemployable in the industry. This would potentially have a deterrent effect on corporate insiders who are often unaffected by the imposition of large corporate fines. Additionally, under the Park Doctrine, the FDA has the ability to bring misdemeanor charges against high-level corporate officers who were in a position to have corrected or prevented violation of the Federal Food, Drug, and Cosmetic Act (that is, ‘responsible corporate officer’) [58,59]. Vigorous application of the Park Doctrine would target high-level officers who are normally harder to prosecute and typically escape liability. Finally, where the improper marketing poses a serious threat to public health, the FDA could require companies to conduct a corrective marketing campaign to fix the misimpression the improper marketing created and formally disavow the erroneous marketing messages the company previously delivered [60]. Corrective actions could be costly and could erode the revenues reaped by fraudulent marketing.

## Conclusions

Scientific journals are intended to serve as a reliable conduit of valid evidence required for healthcare decisions. Evidence of a drug’s effectiveness for off-label indications should be considered a public health commodity [4]. Therefore, if a clinical trial is conducted not to answer a scientific question but to implement a publication strategy or further a marketing goal, it should be disclosed to the peer reviewers, journal editors, and explicitly described in the manuscript. Such disclosure will encourage critical assessment of potential reporting biases in manuscripts of trials for which publication decisions are made by a company’s marketing personnel, based not on the advancement of science but rather on a company’s marketing interests. Other policy options such as mandating registration of all trials, making trial protocols publicly available, and enabling access to trial data can help mitigate the impact of biased reporting on the integrity of the scientific record. Further research should address how frequently pharmaceutical companies employ a publication strategy as a basis for conducting clinical trials, examine the impact of FDA guidance on drug companies’ marketing practices involving the use of publications in medical journals, and quantify the impact of misrepresentation of scientific facts on prescription behavior and on patient outcomes.

## Competing interests

KD served as an expert witness for the plaintiffs’ lawyers in litigation against Pfizer that provided several source documents used for this study. Funds that would have been paid to KD for serving as an expert witness were donated by the plaintiffs’ attorneys to Johns Hopkins for use in scholarly endeavors related to reporting biases. SV was paid by the plaintiffs’ attorneys in litigation against Pfizer for assistance provided to KD for her work as an expert witness. SV’s work on this manuscript and other ongoing research is being supported using the fund established at Johns Hopkins. IR, PG, and TG are affiliated with Greene LLP, a law firm that represents and has represented various clients in litigation against Pfizer, including the cases in which KD served as an expert witness and other cases referred to in this manuscript. The firm has earned legal fees from a case against Pfizer in the past, and the firm is currently involved in other litigation that may result in additional fees.

## Authors’ contributions

Attorneys for the plaintiffs (PG, IR, and TG) provided internal company documents to KD for preparing her report related to reporting biases. Over the course of preparation of the report, and as part of the subsequent research we are conducting, KD and SV requested, and received, further information from plaintiffs’ attorneys related to study conduct, dissemination of findings, and internal discussions about related clinical trials of gabapentin. All authors reviewed all documents used for this study. KD and SV extracted data and assessed the documents for reporting biases, “spin,” and journal circulation. PG, IR, and TG provided insights that facilitated examination of the scientific issues in the context of marketing goals. All findings and interpretations were discussed and agreed among all authors. SV wrote the first draft of the manuscript and KD, PG, IR, and TG critically revised and edited subsequent versions of the manuscript. All authors read and approved the final manuscript.

## Supplementary Material

Additional file 1 **Provided as “Additional file.pdf” in a PDF format.** This file provides the full-text copies of documents for all extracts presented as figures in the manuscripts. Click here for file
