# Supplementary material for: Implementation of a publication strategy in the context of reporting biases. A case study based on new documents from Neurontin® litigation
Source: Trials. 2012 Aug 13;13:136. doi: 10.1186/1745-6215-13-136 (PMC3439687; doi:10.1186/1745-6215-13-136)
Supplement: Additional file 1 — Provided as “Additional file.pdf” in a PDF format. This file provides the full-text copies of documents for all extracts presented as figures in the manuscripts. [file 1745-6215-13-136-S1.pdf]

This Additional file or Online Supplement is provided along with a manuscript submitted by Vedula SS, Goldman PS, Rona IJ, Greene TM, Dickersin K. Implementation of a "publication strategy" in the context of reporting biases. A case study based on new documents from Neurontin(R) litigation.

Online Supplement Figure 1. Extract from marketing assessment for Neurontin® in migraine prophylaxis showing recommendation to conduct publication studies.
Names, signature and contact information of individuals have been blocked out by us such that only the initials can be seen.

Morris Plains, NJ

PARKE-DAVIS
People Who Care

Memorandum

To: DISTRIBUTION

Date: July 31, 1996

From: J[redacted] B[redacted] (PD, Product Planning, Morris Plains, NJ USA)

Subject: ***Neurontin® Marketing Assessment***

Enclosed is the final version of the Marketing Assessment for Neurontin® in migraine prophylaxis which includes the recommendations approved at the last NPC meeting. The decision is to conduct only publication study(ies) in the U.S. due to the current patent situation in the U.S., limited use of anticonvulsants in the EC, and favorable pre-clinical results in analgesia seen with CI-1008.

The results, if positive, will therefore be publicized in medical congresses and published in peer-reviewed journals.

[redacted]

J[redacted] B[redacted]

JTB:nrb
mktg-ass.neu

Enclosure

V082737

CONFIDENTIAL

WLC_FRANKLIN_0000081255

Online Supplement Figure 2. Extract from marketing assessment of Neurontin® in nociceptive pain showing recommendation to use trial data for publication only

1. INTRODUCTION

Purpose

The purpose of this document is to assess the potential of a Neurontin combination product for use in pain. While a variety of possible combination products have been discussed, this brief assessment will focus mainly on Neurontin in combination with an NSAID and narcotics.

2. OBJECTIVE

The concept of a Neurontin combination product was originally considered as a result of a PD US initiated working group, exploring options for patent extension. This was further supported by a number of neuropathic pain thought leaders as they have noticed a synergistic effect when gabapentin is administered concomitantly with amitriptylene. However, due to the development timelines of potential Neurontin combination products and the clinical development of pregabalin (and potentially darbufelone), the main objective is no longer Neurontin patent extension. Rather, the objective is to create a portfolio of products for the treatment of a broad spectrum of pain syndromes ranging from moderate acute to severe chronic pain.

Preclinical Rationale

Animal data for a gabapentin-naproxen sodium combination demonstrated a synergistic effect over gabapentin or naproxen sodium alone, in a rat model of hyperalgesia. Preclinical data also suggests that gabapentin may provide a GI protective effect when combined with indomethacin over indomethacin alone. This would provide additional benefit to the NSAID combination product beyond potential superior efficacy. Additional animal data reported in the literature (Shimoyama, 1997) also established a synergistic effect of gabapentin combined with morphine in rat pain models.

Clinical Rationale

A synergistic effect with morphine has also been demonstrated in a single dose 4-way crossover study in healthy human volunteers (placebo vs. gabapentin vs. morphine vs. combination gabapentin/morphine). The analgesic effect was evaluated by pain threshold time and pain tolerance time. Major findings were (1) gabapentin vs. placebo showed no significant effect difference, (2) morphine vs. placebo showed a clear significant effect difference and (3) the combination of morphine/gabapentin was significantly more effective than morphine alone.

Online Supplement Figure 2. Continued.

A pain market segmentation strategy has previously been implemented successfully by Syntex with Naprosyn and Anaprox. Naprosyn was targeted for OA/RA, while Anaprox was targeted for sports injuries. Detailing for each compound was based on the respective physician audience (e.g., rheumatologists for Naprosyn, emergency room physicians for Anaprox).

~ PAIN PORTFOLIO ~			
**Neurontin**	**Pregabalin**	**Neurontin + NSAID**	**Neurontin + Opioid**
Neuropathic Pain (Europe)	Chronic Pain	Acute Pain	Severe Pain
Approval: 2000	*Approval:* 4Q 2001	*Approval:* 4Q 2001	*Approval:* 2003

Pregabalin combinations would be introduced starting in 2006 as line extensions to pregabalin, potentially replacing Neurontin combinations with newer, more potent or safe components.

Clinical Development Issues

Potential areas of concern that have been raised include:

- ***Formulation*** – There may be compatibility issues with naproxen. Furthermore, if the dose needed is relatively high, size of the tablet/capsule may become problematic. Additionally, the lactam content of Neurontin may be affected by such a combination. As well, there is a need to develop two dosing strengths. Finally, a BID dosing program is preferred.

- ***Dose ratio*** – The animal data suggest a dose ratio of 1:1 on a mg to mg basis is the optimum formulation. It is uncertain if that will hold true for human trials.

- ***Timeline*** – The combination product would require a full development effort to develop a full regulatory dossier, although it may require less toxicology work since existing data in the public domain may suffice.

- ***Choice of NSAID*** – While naproxen is a leading compound in the US, in Europe diclofenac is clearly the dominating NSAID. However, based on the fact that only the UK may represent feasible commercial potential outside the US, there may not be an issue.

- ***Patent*** – Parke-Davis has filed patents for all potential combination products for both Neurontin and pregabalin.

Regulatory Strategy

Although a combination pain product NDA/MAA would be unique for Parke-Davis, numerous examples of such products exist in the US, including Arthrotec and Vicoprofen, , and a plethora of combination analgesics in Europe.

Online Supplement Figure 2. Continued.

Our primary objective is to demonstrate better efficacy with a Neurontin + naproxen sodium combination product than either product alone with doses lower than what is generically available for the treatment of low back pain, sprains, and strains. Hence, clinical trials should be powered for the primary endpoint, efficacy. Secondary endpoints should include GI tolerability, etc., although not powered to make a comparative claim. Such secondary data would be for publication only.

6. PRIMARY MARKET RESEARCH

Top Line Report for Combination Product

Primary market research was conducted with primary care physicians, orthopedic surgeons, and neurologists to initiate the process of understanding the acute and chronic pain (e.g. OA) market and to obtain a preliminary assessment of interest in Neurontin combination products for pain, in particular, a Neurontin/NSAID combination.

The interest expressed in the new product concept for the NSAID/anti-convulsant combination was at a level in this study that would indicate a recommendation to continue exploring the potential of such a compound.

In general, most respondents in this study were not satisfied with the available pain products on the market. Physicians in this study were using a multitude of products to treat both acute and chronic pain. For acute pain, these products included Toradol, Cataflam, ibuprofen, Darvocet, hydrocodone, Ultram, Tylenol #3, codeine, Tylenol, Relafen and others. For chronic pain control respondents mentioned using Percocet, Darvocet, Ultram, Neurontin, Elavil, codeine, Lortab, Lorcet, Tegretol, Esgesic, and others.

Combination therapy was most frequently used for back and neck pain, and post surgery pain control. Frequent combinations mentioned included, ibuprofen/Darvocet, Advil/Vicodin, Naproxen/Daypro, ibuprofen/Toradol, Neurontin/Tegretol and others.

Combination therapy was used most frequently for acute pain in this study, but was also used in chronic therapy. The hesitancy in chronic therapy stemmed from unknown outcomes of long-term use of combination therapy, and the belief that the fewer the drugs, the better for the patient.

As could be expected, GI side effects for the NSAIDs, and addictive potential for the narcotics were the greatest concerns for the use of pain medications. CNS side effects and lack of reliable efficacy of the anti-convulsants, and the risk of tachyphylaxis with some pain meds were also mentioned as concerns.

New Product Concept

In general, the New Product concept was well received. Neurologists gave the least favorable review of the product based mainly on the inability to titrate the two compounds.

Pfizer_MPierce_0000835

Online Supplement Figure 3. Internal company document illustrating role of Medical Action Communications, a medical writing company, in developing key messages based on a branding guide for Neurontin

4.0	(eg, Aricept, Zoloft, Relpax) it was suggested that this may not be a global Pfizer policy but that individual teams had different approaches to this issue. It was suggested that this should be discussed in more detail internally with the Neurontin team before any policies were put in place for the Neurontin team. The general agreement is that Pfizer employees should not be 1st or last authors and a ratio of >/= 3/1 (outside to Pfizer) in authors should be maintained.	KK
5.0	**Key Message Development Update:** MAC updated the PSC regarding the development of key messages, indicating that the branding guide had been received and that it was anticipated that the draft key message list would be circulated to the team by 25-July. The team will then be asked to comment on the key message list and provide feedback by 1-August.	MAC<br>PSC members
	**Bibliography Database search terms:** The team reviewed the draft list of key search terms that was developed. This list represents the major search categories that will be included in the database, as well as a preliminary list of searchable terms within each category. The team was asked to review this list and submit and comments or questions to SV by 30-July.	PSC members
6.0	It was pointed out that one category of search term was not included in this list--key words. This category is currently being developed and will include terms from many of the other categories, as well as other terms of key relevance to the team (eg, competitor product generic and brand names). This expanded list will be submitted to the PSC for comment by 25-July.	MAC
	**Current Neurontin publications:** The status of two current publications was discussed:<br>*1) Gabapentin vs Lamotrigine, monotherapy in epilepsy; Lancet publication.*<br>The manuscript was discussed with the two primary authors on 18-July and there was a question regarding the statistical analysis The manuscript is awaiting submission based on Pfizer approval.<br><br>RG asked for clarification regarding the composition of the patient population for this study (number of partial vs generalized siezures). The team was informed that the study contains 20% patients with generalized seizures. RG recommended that it may be useful to consider a secondary publication that reports on a sub analysis of patients with partial seizures to demonstrate the significantly greater clinical efficacy in this area The team agreed that this might be a worthwhile pursuit and additionally suggested that there may be other sub analyses that are worthwhile considering for this study once the primary data is published. MAC agreed to work with the team to review the study and identify appropriate targets for sub analysis (eg, dosing schedule, time to exit, etc). The PSC approved the manuscript for submission.	MAC/PSC members<br>KK

Action Report: 18-July -01 3

Pfizer_RGlanzman_0044636

Online Supplement Figure 4. Standard operating procedure (SOP) specified that "affiliate-driven manuscripts" should be submitted for review by Neurontin Publications Sub-Committee (NTN PSC) to ensure the content is consistent with "current product messages"

SOP for affiliate-driven manuscripts

- In April 2002 a memo was sent to all affiliates requesting that affiliate-driven manuscripts be forwarded to the NTN PSC for review. The reasons for the request are listed below.
  - –To review all manuscripts to ensure that they are in-line with current product messages and areas of interest.
  - –To avoid publication delays by providing assistance with translation of manuscripts into grammatically correct English.
  - –To identify manuscripts that are a global priority.
- The PSC agreed to assign a lead reviewer to each manuscript received. The reviewer would review and forward thoughts on the level of PSC involvement needed using the form developed within 1 week of the PSC meeting. This would then be communicated back to the affiliate.
- The goal of this process was to provide feedback to the affiliate as quickly as possible as well as to keep NYHQ aware of what areas affiliates were pursuing.

NEURONTIN publication
(gabapentin) planning 2002

Pfizer_LeslieTive_0091420

Online Supplementary Appendix Figure 5. Peer review comments from two journals to which findings from Study 945-224 were submitted.

Submission of 945-224 results to Diabetic Medicine

CONFIDENTIAL

Pfizer_LeslieTive_0020880

Online Supplement Figure 5. Continued.

31.03.2003 09:46Roder, Beate

R[redacted] B[redacted]

**Von:**	ScholarOneMailer@ScholarOne.com
**Gesendet:**	Montag, 13. Mai 2002 14:17
**An:**	b[redacted]r[redacted]@pfizer.com
**Betreff:**	Diabetic Medicine DME-2002-00105

Re Gabapentin in painful diabetic neuropathy: a randomised, double-blind, placebo-controlled study

Dear Dr. R[redacted]

Thank you for submitting your paper to Diabetic Medicine. I regret that it has not been accepted for publication as it stands.

I attach the reports of the associate editor and two reviewers. You can see that they find your manuscript of interest but have raised significant concerns which would need to be addressed.

If you feel able to respond to the reviewers' comments :hen we will give careful consideration to a resubmitted paper. I should emphasise that we are certainly not guaranteeing acceptance at this stage. A decision will be based on whether you can meet the reviewers' concerns.

Your responses to each point made by the reviewers should be made at http://dme.manuscriptcentral.com through your Author Centre by clicking the appropriate button. Please then submit your revised manuscript by clicking its title. You will prompted to upload the file(s).

Any revised paper should be submitted to the Diabetic Medicine site within two months of your receipt of this letter.

Thank you for submitting to Diabetic Medicine.

Yours sincerely.

Dr S[redacted] H[redacted]
Editor, Diabetic Medicine

Associate Editor comments:

In this multicentre controlled trial Reckless and coworkers evaluated the efficacy and safety of gabapentin (600, 1200, 2400 mg/day) compared with placebo treatment in 325 diabetic patients with painful neuropathy over 7 weeks. A subgroup of 67 patients received the drug in a subsequent 4-month open-label period. After 7 weeks the primary outcome measure (weekly mean pain score) was not improved in favor of gabapentin. In contrast, several secondary endpoints did show improvement in excess of placebo. The authors conclude that while gabapentin did not demonstrate significant effects on the primary endpoint, the improvements of some secondary endpoints indicate an overall benefit from gabapentin in painful diabetic neuropathy. This manuscript has been reviewed by two referees and a statistical advisor. Both reviewers felt that although this trial deals with an important problem in diabetic patients, they identified numerous points of critique regarding data analysis and inter! pretation that need to be carefully addressed. As stated by the statistical advisors, the quality of the statistics appears to be poor, and hence, the conclusions are not justified. In summary, this large controlled trial addresses an important area of patient care. However, as the present study could not demonstrate significant effects on the primary endpoint, it contrasts with the results of a previously published US trial. The authors are advised to perform an appropriate statistical analysis which should allow them to draw a less biased interpretation given the evidence indicating that gabapentin had no effect on the weekly mean pain score.

Online Supplement Figure 5. Continued.

31.03.2003 09:46[redacted]B[redacted]
Reviewer comments:

Reviewer 1 Comments:

This is an important paper about a common problem, painful diabetic neuropathy, but needs rewritting. I have several concerns about the paper. First, I believe that the stat section should use the Bonferroni correction, given the multiple comparisons made on the same data set. This would require a final p value of p<0.01 or even p <0.005, depending on the calculation of the correction, to be considered significant. With this redo of the data, it is probable that NO statistical measures were postive. Thus, the trial would be considered a failure and the paper rewritten accordingly. Second, the issue is why this trial failed when the US trial of 3600 mg was so positive. This is an important issue esp if the hightened expectations of patients and MDs contributed to the large placebo effect. This needs more explanation along with other possible factors. Third, even with the high placebo effect the negative trial does not fit with the US trial as the mean effective dose in th!
at trial was 1800 mg. Is there no dose response curve? Luckily, the FDA is not asked to consider this in looking at Neurontin. Third, there are many areas of company bias that need elimination. For example, only carbemazipine is discussed but many other drugs have favorable trials.

Reviewer 2 Comments:

GBP in PDN by R[redacted]J et al submitted to Diabetic Medicine

This study investigated the efficacy of gabapentin (GBP) at 3 doses in relieving the pain of painful diabetic neuropathy when compared to placebo. In addition to the primary outcome of pain ratings authors used secondary outcomes such as sleep interference and quality of life (QoL) assessment tool. After 7 weeks of double-blinded treatment it was established that GBP was not different from placebo in its primary outcome. Only significant effects of GBP were: the middle 1200mg/day dose as clinical global impression of change and in some QoL measures, and at 1200mg/day and 2400mg/day dose in alleviating sleep interference due to pain. The interpretation was that the placebo effect was so high due to expectation on the part of physicians and patients.

There are many aspects of this study that need to be addressed.

The results are intriguing in many regards. The most "effective" dose, in the sense that it showed significant results in secondary outcomes was the middle dose, which was also the dose that was associated with least adverse events among GBP doses and with the lowest the dropout rate, even less than placebo, though apparently that was not statistically significant. But lower rate of adverse effects alone could not provide the explanation for middle dose to be "more effective" than the higher 2400mg/day dose since published literature (US studies) demonstrated that most patients were able to tolerate higher, 3600mg/day dose with clearly superior pain relief. Is there a president in the literature where the middle dose was more effective than the higher dose? I am not aware of any.

The issue of the highest dose selected for this study brings us to the statement on page 11, second paragraph, last sentence: "The underlying assumption was that pain relief obtained from at least 1 of the doses used in this study (600, 1200 and 2400mg/day) would be in the same order of magnitude as that seen with 3600mg/day gabapentin in the United States study" - this statement defies logic since only one dose, 2400mg/day, was the only one that was higher than what was suggested by US study to be effective dose of 1800mg/day or higher. Were the authors looking for the least effective dose? If so, that should be stated as such. Also, what was the reason that authors did not use 3600mg/day when that was shown to be safe and effective dose, even if they were looking for least effective dose? Please explain.

Most puzzling of all was the effect of the lowest dose of 600mg/day fared much worse in all outcomes including adverse events, even worse than placebo. Authors did not provide information whether any of that was significant? Did these patients experience all the adverse effects without any benefits so that is why they came out worse than any other group? Is small dose GBP pro-nociceptive? Please explain.

Authors are requested to present the pain rating (means) data over the duration of the study as line graph. It should be instructive.

It is unlikely that adverse events were the cause for such a high placebo response rate. However, one way to monitor the possible influence of unmasking investigators

Online Supplement Figure 5. Continued.

31.03.2003 09:46R[redacted]B[redacted]

are advised to ask each of their patient at the conclusion about their impression what treatment they thought they received. Was this done in this study?

In the Patients and Method section under Safety Evaluation it is stated that physical examination, including sensory neurological examination was performed but symptoms and signs which define any neuropathic pain, including painful diabetic neuropathy was not analyzed and discussed as one of the outcomes. That is most surprising since a few of the participating authors are recognized experts in the area of neuropathic pain. Please provide the information about neurological sensory examination. This may especially be important for the group that did the worst, 600mg/day.

Statistical Advisor Comments:

On page 13 it is not correct to report both endpoint scores and change in pain score. The same applies to sleep interference scores. Repeated measures analysis of variance should be used to analyse data collected sequentially.

What was compliance? Were table counts done?

In sleep interference score were 1200 and 2400 mg groups combined? Should reported measures ANOVA of 4 groups have been done and then a predetermined tend test across 3 levels of medication?

Figure 3 should be cumulative block charts. Why were 'very much' and 'much improved' groups combined? A chi-squared test for trend should have been done, not ANOVA, this is ranked categorical data, not continuous data.

p = 0.0414 (page 15) cannot really be considered significant in the light of the number of tests performed. Was a Bonferronni correction discussed?

Tests for trend should be done on data in table 5.

What were the nervous system adverse events?

Online Supplement Figure 5. Continued.

Submission of 945-224 results to Diabetologia

CONFIDENTIAL

Pfizer_LeslieTive_0020840

Online Supplement Figure 5. Continued.

15/11/2002 16:12 +43-1-40400-2728 DIABETOLOGIA S. 01

Diabetologia

Journal of the European Association for the Study of Diabetes (EASD)
Editor-in-Chief
W[redacted] W[redacted] M.D, Vienna
Tel: 0043 1 404002727 Fax: 0043 1 40400 2728
e-mail: diabetologia@akh-wien.ac.at

Dr. B[redacted] R[redacted]
Pfizer GmbH

Mooswaldallee 1
79090 Freiburg
Germany

Editorial office
P.O. Box 27
1097 Vienna,
Austria

Fax-No.:+ 49 761 518 3073

14th November 2002

Re:Diabet/2002/000754 J[redacted] R[redacted] et al., ' *Gabapentin in painful diabetic neuropathy: a randomised, double-blind, placebo-controlled study* '
Received: 22nd October 2002

Dear Dr. B[redacted] R[redacted]

Your above-referenced manuscript has been read by two experts in the field but I regret to inform you that we are not able to offer publication in *Diabetologia*. This decision is based on the evaluation of the referees, whose reports are enclosed, as well as on priorities set by the Editorial Board.

Unfortunately we are not able to publish all the manuscripts that receive positive comments. The severe competition for space in the journal forces us to reject quite a few manuscripts which are of sound scientific quality but which are not allocated top priority for publication in *Diabetologia* by the editorial board.

I hope that the referees' evaluations will be helpful if you plan to revise the manuscript for submission to another journal.

I am sorry that I could not be more positive on this occasion but hope we can look forward to other contributions from you in the future. Thank you very much for allowing us to review your manuscript for *Diabetologia*.

Yours sincerely,

W[redacted] W[redacted] M.D
Editor-in-Chief

CONFIDENTIAL

Pfizer_LeslieTive_0020841

Online Supplement Figure 5. Continued.

15/11/2002 16:12 +43-1-40400-2728 DIABETOLOGIA S. 02

Diabetologia

Manuscript reference: Diabet/2002/000754 J[redacted] R[redacted] al
Received: 22nd October 2002
Date sent: 30th October 2002 Referce code: A
Referee recommendation (Please do not give advice as to acceptance/rejection on this form)

The authors report a randomized trial assessing the efficacy and tolerability of Gabapentin in painful diabetic neuropathy.

1. I am not sure as to the need for a trial such as this as there are already a number of controlled trials in the literature looking at Gabapentin in diabetic neuropathy.
2. The authors, indeed, state that they wanted to confirm previous positive findings, but then seem to forget that the mean dose for adequate pain relief is previous trials was much higher than the first two doses in this present trial.
3. What is really needed is comparative trials of Gabapentin versus other known treatments for diabetic neuropathies. It is surprising, therefore, that the authors fail to refer to the previous trial of Gabapentin versus Amitriptyline published by Morello et al., in the *Archives of Internal Medicine* two years ago.
4. The entry criteria for neuropathy are poorly stated. Table 1 is completely unacceptable and looks like a table extracted directly from a pharmaceutical company protocol.
5. The authors state that neuropathy is defined according to the San Antonio criteria. Did they really do detailed autonomic and peripheral nervous function testing?
6. If they only permitted analgesa in the screening and trial period with paracetamol, I am concerned as to whether these patients really did have significant painful neuropathy.
7. Why did the authors elect to do a 7-week study which seems rather short?
8. My concern that this is a pharmaceutical house prepared paper seems to be confirmed by my observation that reference 6 is even listed as a Parke-Davis study!

CONFIDENTIAL

Pfizer_LeslieTive_0020842

Online Supplement Figure 5. Continued.

15/11/2002 16:12 +43-1-40400-2728 DIABETOLOGIA S. 03

Diabetologia

Manuscript reference: **Diabet/2002/000754** J[redacted]R[redacted] al
Received: 22nd October 2002
Date sent: 24th October 2002 Referee code: B

Referee recommendation (Please do not give advice as to acceptance/rejection on this form)

Reckless et.al. provide the results of the largest randomised, double blind, placebo controlled study assessing the effects of Gabapentin on painful diabetic neuropathy to date.

The data from this trial clarifies some misconceptions of the supposed efficacy of Gabapentin and enables clinicians treating patients with painful diabetic neuropathy to evaluate the potential of this drug.

Important lessons to be learnt from this study:

1) For pain scores Gabapentin is not effective.
2) If it is effective then 600mg Gabapentin does not work which should allow us to start at a more effective dose.
3) There is no apparent dose response curve as there was no difference between 1200 and 2400mg. Perhaps 1200mg may be the optimal dose but even this is not significantly different from placebo.

No change in VAS or PPI normally gold standard measures of therapeutic efficacy in clinical trials of pain.

There is a benefit on sleep scores, is this as a consequence of the side effect of somnolence?

Surprisingly some components of the SF-36 QoL improved but not compared to placebo and in the lowest 600mg and highest 2400mg dose and not with 1200mg.

Does this suggest other, none analgesic related benefits of Gabapentin.

Why did only 67 patients continue in the open-label study? If the drug is truly effective would you not expect more patients to have gone to open label.

We need some details on proportion that were on therapy for their pain prior to study entry and groups of medications they were on (tricyclics etc.)

Dizziness and somnolence the most commonly reported adverse events occur in a large proportion of patients (24.3%) confirming day to day clinical experience.

What is the NNT for this study?

CONFIDENTIAL Pfizer_LeslieTive_0020843
